# Commentary: Life is unfair, and so are racing sports: some athletes can randomly benefit from alerting effects due to inconsistent starting procedures

**DOI:** 10.3389/fpsyg.2016.00119

**Published:** 2016-02-09

**Authors:** Edwin S. Dalmaijer, Beorn G. Nijenhuis, Stefan Van der Stigchel

**Affiliations:** ^1^Department of Experimental Psychology, University of OxfordOxford, UK; ^2^Department of Experimental Psychology, Utrecht UniversityUtrecht, Netherlands

**Keywords:** alerting, temporal expectancy, foreperiod, racing, sports

In some racing sports, regulations require a variable time between the referee's “*Ready*” cue, and the starting shot. Psychological experiments demonstrate that the length of a pause between such a non-spatial cue and the following signal affects the response to that signal: Reaction times are lowest after an optimal interval of 500 ms, and progressively increase as the interval increases to several seconds (Posner and Boies, 1971; Sanders, 1975). This phenomenon is attributed to a short-lived boost in arousal, and is referred to as the *alerting effect*. In a recent *Perspective* article in *Frontiers in Psychology*, Dalmaijer et al. (2015) argue that alerting effects could allow athletes who start with shorter ready-start intervals (RSIs) to respond quicker to the starting shot. They support their claim with a correlation between RSIs and race times from the 500-m speed-skating event at the 2010 Winter Olympics (Figure 1A), and suggested temporal variability should be removed from starting procedures in racing sports to avoid biased competitions.

**Figure 1:**
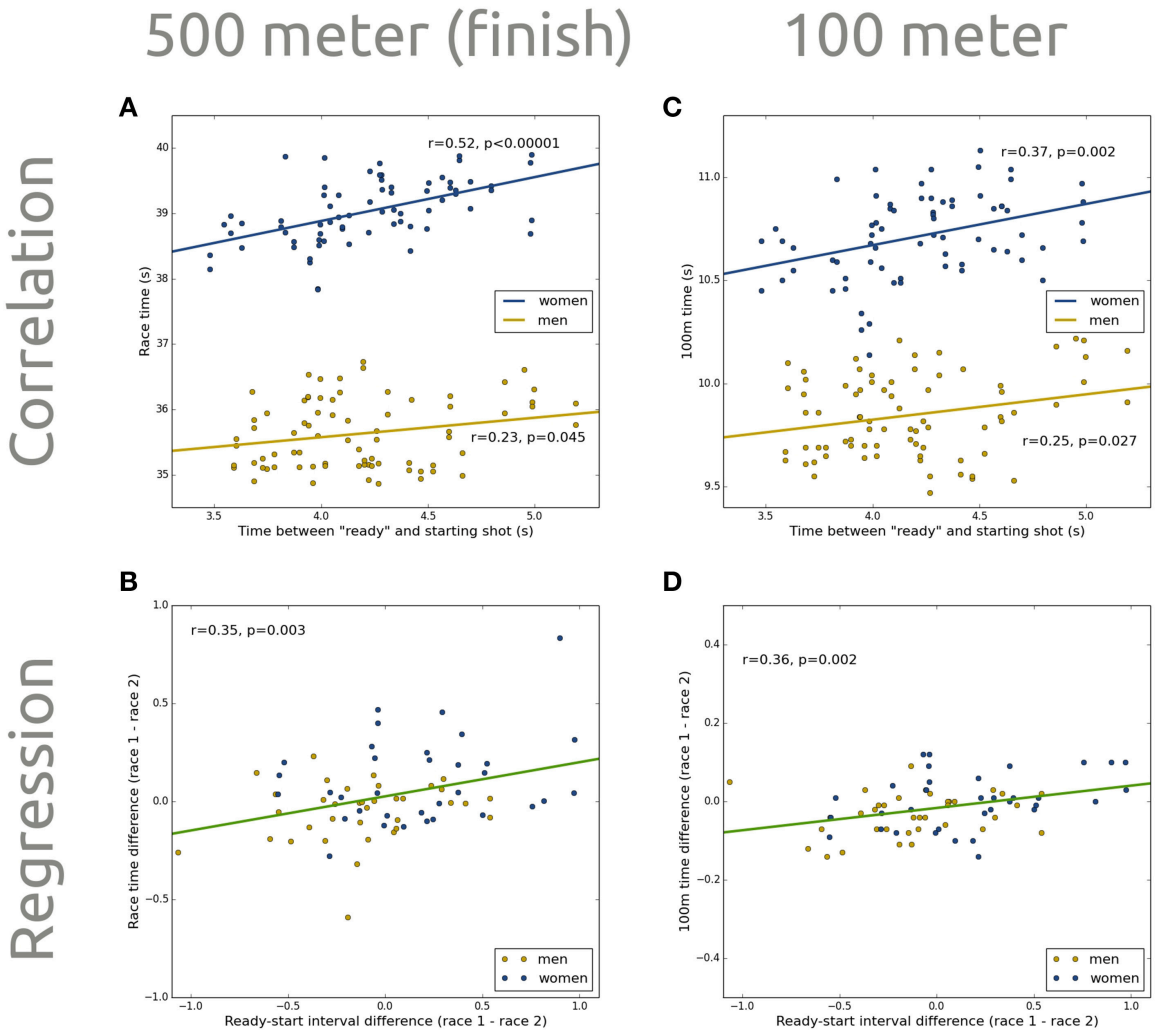
**The time between a referee's “Ready” cue and the starting shot is variable, and correlates positively with speed-skaters' times at the finish (A), and 100 m into their race (C)**. This is true for men (yellow) and women (blue). Group-level (men and women combined) within-skaters differences in ready-start interval predict within-skaters differences in times at the finish **(B)** and 100 m into their race **(D)**.

News media reported on the study (Daily Mail Online, 2015; Huffington Post, 2015; Süddeutsche Zeitung, 2015). The resulting public attention sparked debates amongst speed-skating enthusiasts and professionals, revolving around two questions: Do quicker athletes somehow produce shorter RSIs, and should the effects of RSIs have been tested earlier into a race? Here, we address the raised concerns with additional data and analyses.

## Are better skaters quicker to start?

Most comments on Dalmaijer et al. argued that correlation does not imply causation to support speculative alternative explanations: Referees could be more nervous with higher-profile athletes, or better athletes could be quicker at assuming their starting positions. Although, no alternative is supported by scientific literature, they would produce the observed correlation between RSI and race time.

Borghans (2015) re-analyzed Dalmaijer et al.'s data, capitalizing on how each speed-skater participated in two races. These are independent (each with different RSIs, lane positions, ice conditions, and opponents), making it possible to correlate RSIs of one race with finishing times of the other. There is a significant correlation between one race's RSI and the other race's time for women (*r* = 0.40, *p* < 0.001), but not for men (*r* = 0.22, *p* = 0.065). Borghans argued this demonstrates that better skaters are more consistent, and quicker to start.

Although clever, this approach confounds the effects of skater consistency and RSI. Athletes can be *both*: consistent in their starts, and affected by RSIs. Thus there *should* be a correlation between a skater's RSI on one race and their time on the other.

A better approach is to use the *difference* in RSIs between individual's races to predict the *difference* in their finishing times. Even if better skaters are quicker to assume position, the relation between these differences should be in the predicted direction. To capture this direction, but not external influences or moderators, one could treat the referee as an experimenter who changes the RSI between two races of the same athlete. In this framework, the RSI *difference* could be regarded as an independent variable, which would allow causal inferences about its effect on race time differences.

We performed a linear regression on the differences in RSI and race time between the first and the second race of each athlete. One athlete did not participate in both races, and four were excluded for (nearly) falling, leaving 70 samples. RSI difference was a significant (*p* = 0.003) predictor of race time difference, explaining 12% of the variance (Figure 1B). One additional second of RSI difference led to 174 ms additional race time difference. Thus, RSIs have an effect on race times, even when accounting for potential confounds.

## Do ready-start intervals affect 100-m times?

Another prevalent question was whether the effect of RSIs on skaters' performance would be present earlier in races. In 500-m speed skating competitions, times are registered 100 m into the race. A current professional skater expressed that he thought to be highly consistent in his 100-m time, and doubted RSIs would affect this (NOS-Dutch Broadcasting Foundation, 2015a,b). In addition, some noted that if Dalmaijer et al.'s results were due to reduced response times at the start, 100-m times would be a more direct index than finish times.

An enthusiast kindly supplied us with the 100-m times from the competition analyzed by Dalmaijer et al. We analyzed them exactly as the 500-m times, and found a significant correlation between RSIs and 100-m times for men (*r* = 0.25, *p* = 0.027, *N* = 77) and women (*r* = 0.37, *p* = 0.002, *N* = 70; Figure 1C). In addition, within-skater differences in RSI were a significant (*p* = 0.002) predictor of within-skater differences in 100-m times, explaining 13% of the variance (Figure 1D). One additional second of RSI difference led to an additional 59 ms of 100-m time difference.

## Future directions

Our results indicate that RSIs affect 100-m and finishing times. RSI differences can explain roughly the same variation in 100 and 500-m time differences, and the effect scales from 59 ms at 100 m to 174 ms at 500 m. This is surprising, as Dalmaijer et al.'s (2015) alerting hypothesis concerns athletes' reaction times at the start. During a race, effects of other factors (e.g., athletes' qualities) should account for an increasing proportion of the variance. Therefore, alerting could only be part of the story.

Some suggested that athletes could be sensitive to their own reaction times. Starting slower could lead to reduced motivation or concentration throughout the race, thereby reducing performance.

Others hypothesized that longer RSIs could induce a subtle increase in muscle fatigue. Previous research has shown that muscle fatigue builds up from the onset of exertion, subtly reducing force output within seconds (Bigland-Ritchie et al., 1983; Kent-Braun, 1999). However, it is unclear whether this could have an effect on athletes' performance.

## Conclusion

Although Dalmaijer et al.'s (2015) analysis could have been more elegant, the theoretical framework was valid, and the claims hold up in more stringent analyses. We demonstrate that differences in ready-start intervals predict differences in finishing times. Although the underlying mechanisms should be investigated in future experimental research, sports unions would be wise to exercise caution with the current starting procedures.

## Author contributions

All authors conceived the idea. ED collected and analyzed the data. ED drafted the manuscript, and BN and SV provided critical comments.

### Conflict of interest statement

The authors declare that the research was conducted in the absence of any commercial or financial relationships that could be construed as a potential conflict of interest.
